# Intravital microscopic observation of the microvasculature during hemodialysis in healthy rats

**DOI:** 10.1038/s41598-021-03681-2

**Published:** 2022-01-07

**Authors:** B. G. H. Janssen, Y. M. Zhang, I. Kosik, A. Akbari, C. W. McIntyre

**Affiliations:** 1 Department of Medical Biophysics, Western University, London, ON Canada; 2 Kidney Clinical Research Unit, Lawson Health Research Institute, London, ON Canada; 3 Imaging Program, Lawson Health Research Institute, St. Joseph’s Health Care, London, ON Canada; 4 Robarts Research Institute, Western University, London, ON Canada; 5 Trauma Research Centre, Fourth Medical Center of the Chinese PLA General Hospital, Beijing, 100048 People’s Republic of China; 6 Intensive Care Unit, Tianjin Nankai Hospital, Tianjin, 300100 People’s Republic of China; 7 Kidney Clinical Research Unit (KCRU), London Health Sciences Centre, 800 Commissioners Rd. East, London, ON N6C 6B5 Canada

**Keywords:** Preclinical research, Haemodialysis

## Abstract

Hemodialysis (HD) provides life-saving treatment for kidney failure. Patient mortality is extremely high, with cardiovascular disease (CVD) being the leading cause of death. This results from both a high underlying burden of cardiovascular disease, as well as additional physiological stress from the HD procedure itself. Clinical observations indicate that HD is associated with microvascular dysfunction (MD), underlining the need for a fundamental pathophysiological assessment of the microcirculatory consequences of HD. We therefore successfully developed an experimental small animal model, that allows for a simultaneous real-time assessment of the microvasculature. Using in-house built ultra-low surface area dialyzers and miniaturized extracorporeal circuit, we successfully dialyzed male Wistar Kyoto rats and combined this with a simultaneous intravital microscopic observation of the EDL microvasculature. Our results show that even in healthy animals, a euvolemic HD procedure can induce a significant systemic hemodynamic disturbance and induce disruption of microvascular perfusion (as evidence by a reduction in the proportion of the observed microcirculation receiving blood flow). This study, using a new small animal hemodialysis model, has allowed direct demonstration that microvascular blood flow in tissue in skeletal muscle is acutely reduced during HD, potentially in concert with other microvascular beds. It shows that preclinical small animal models can be used to further investigate HD-induced ischemic organ injury and allow rapid throughput of putative interventions directed at reducing HD-induced multi-organ ischemic injury.

## Introduction

Patients suffering from severe chronic kidney disease (CKD) rely on hemodialysis (HD) for renal replacement therapy. It provides life-saving treatment for kidney failure for around three million people globally, typically consuming 5–10% of total healthcare budgets in the developed world. Quality of life in patients is poor and mortality is exceptionally high; one in three patients will die within a year of starting dialysis, with cardiovascular disease (CVD) being the leading cause of death^1,2^. This huge excess of CV mortality directly results of an elevated prevalence of an underlying CV disease in patients with kidney disease^3,4^. Although HD treatment improves patients' overall health and reduces CVD morbidity and mortality^5^, a significant portion of CKD patients on HD will still be confronted with elevated risks to develop severe cardiovascular complications during and between dialysis treatments. this is further exacerbated by additional physiological stress from the HD procedure itself. More importantly, it has now been recognized that HD acts as an independent risk factor for de-novo and recurrent heart failure^6^, signifying that in a significant portion of HD patients, without any pre-existing cardiac disease, will develop CVD directly resulting from the recurrent HD procedure itself.

HD causes recurrent and cumulative ischemic injury to the heart (driving cardiac sudden death and the development of heart failure)^7^ and other vulnerable vascular beds-resulting in brain injury (cognitive impairment)^8^, liver injury (reduced toxin clearance) and kidney injury (reduced residual renal function)^8^. Multimodal imaging studies during HD have confirmed a reduction in organ perfusion both early in the treatment session (blood contacting the extracorporeal circuit) and later, as a consequence of fluid removal and hypotension^9,10^. These early reductions in tissue perfusion occur before significant circulatory stress has been applied and in combination with observed effects of HD on children and adult patients with acute kidney injury (without a background of CKD), strongly suggest a significant effect on microcirculatory flow during the HD procedure^11–13^.

Microvascular dysfunction (MD) involves the maldistribution of blood flow in the tissue, leaving significant areas under-perfused. MD develops rapidly and can be observed during several pathophysiological circumstances^14–20^, suggesting that MD constitutes a standard feature found in a range of different pathologies. MD is associated with activation of platelets, resulting in the formation of microthrombi reducing effective tissue perfusion^21^. MD is commonly found in critically ill patients, interfering with adequate blood flow to tissue and organs, ultimately leading to irreversible tissue damage and multiple organ failure^22^. Often the heart is separately affected reducing myocardial microvascular perfusion and overall cardiac contractile function^23^, compounding the effects of MD in tissues. Recent observations indicate that dialysis procedure directly leads to a functional reduction of microvascular perfusion throughout the body, while investigations in the cutaneous perfusion, effectively detect HD-induced acute myocardial ischemic injury^24–26^.

Direct experimental visualization and quantification of MD are usually done using intravital video microscopy (IVM), allowing for a detailed visualization and investigation of the microvascular blood flow in surgically exposed tissue in a living animal, and as such, can be used to investigate the development of MD under different pathophysiological circumstances^27–29^.

To our knowledge, no pre-clinical small animal model currently exists that allows for the direct observation of the microvascular perfusion during a HD procedure. The significance of such a model is that it would greatly facilitate the investigation in how changes in treatment procedure, dialyzer design, or new pharmacological treatment strategies, affect hemodialysis and its effects on microvascular perfusion. In this study we describe and present a novel small animal dialysis model and utilize it to demonstrate the negative effects of HD directly on microcirculatory function.

## Materials and methods

### Experimental animals

All experimental animal work described in this study was performed in accordance with the legal guidelines and regulations set by the Canadian Council of Animal Care (CCAC) and approved by the Animal Care and Use Committee (ACUC) of Western University, London, Ontario, Canada. All animal work in the study was carried out in compliance with the ARRIVE guidelines (https://arriveguidelines.org)^30^.

Thirteen male Wistar Kyoto rats (weight approx. 250–300 g, SPF, Charles River, Wilmington, MA, USA) were housed under standard conditions, i.e., water and food ad libitum, 12/12 light–dark cycle at room temperature. Animals were allowed to acclimatize for at least 72 h after arrival at our animal facility before entering the experimental procedures. All were fully anaesthetized with an isoflurane oxygen mixture (induction 4% isoflurane) and free breathing during the experiment. Anesthesia was tapered (2%) to minimize its effects on blood pressure^31,32^.

Body temperature was maintained at 36.5 °C using a rectal probe and an infrared lamp connected to an automated temperature monitor (TCAT-2 Temperature Controller, Physitemp Instruments Clifton NJ). A catheter was placed in the carotid artery and connected to a pressure transducer, to monitor mean arterial blood pressure and heart rate using a rodent blood pressure analyzer (DMSI-400, Micro-Med Inc, Louisville KY USA).

Minimal perfusion with heparinized (2 U/ml) dialysate fluid was used to ensure the carotid catheter’s patency during the complete procedure (approximately 6 h). Pump rate was set to such a level that allowed for a limited inflow of arterial blood into the catheter. Keeping the blood-fluid boundary in a constant position in the catheter, indicated that both blood pressure and pump rate were in close equilibrium with only a minimal amount of fluid being infused.

**Figure 1:**
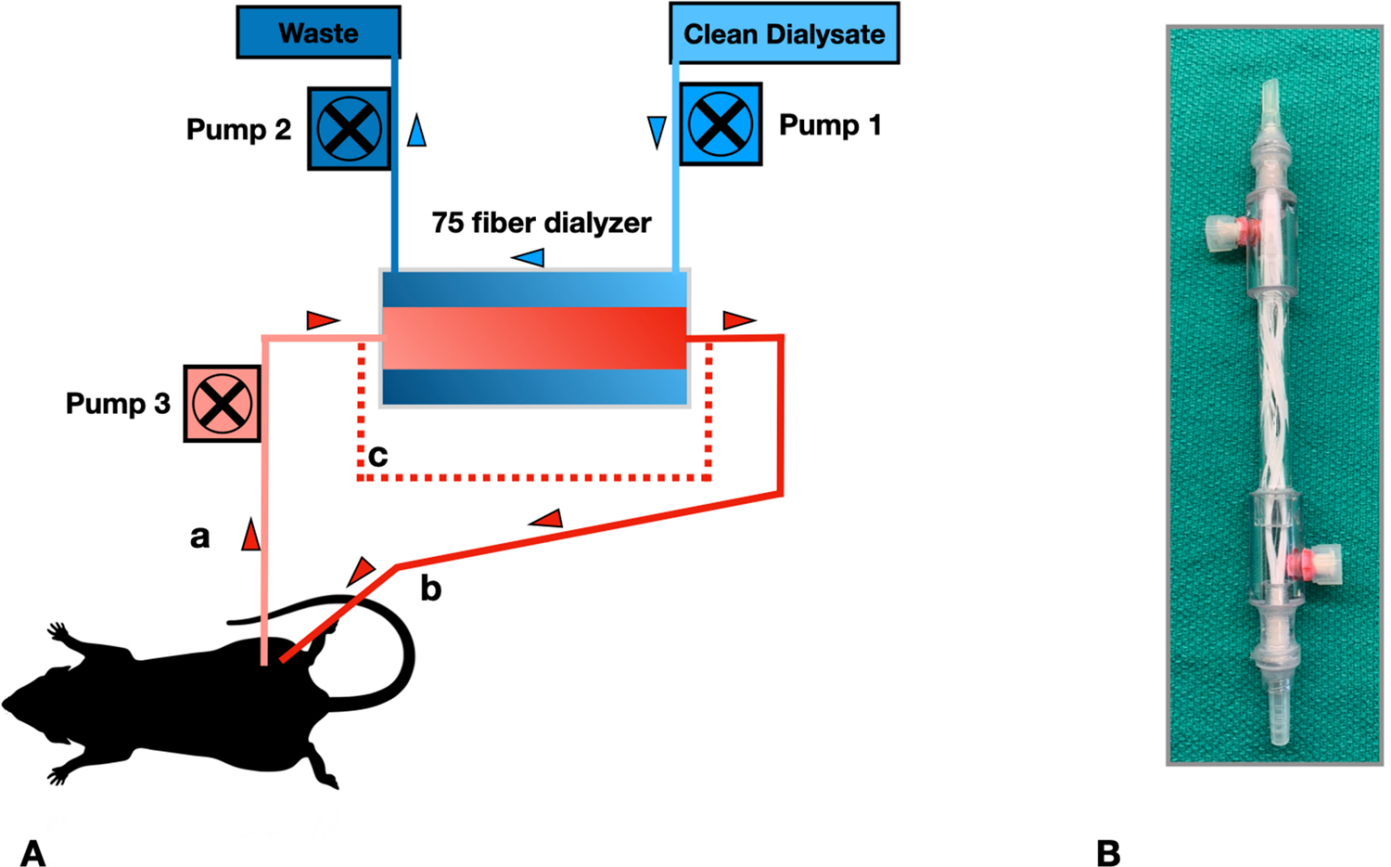
Dialysis setup and mini-dialyzer. (**A**) Schematic overview of dialysis setup. a: catheter left femoral artery, b: catheter left femoral vein, c: the bypass shunt used for sham procedure. Blood pumps, 1 & 2 dialysate pumps. Blood is pumped (pump 3) from the femoral artery through the dialysis unit to the tail vein, while dialysate is counter-currently pumped through the dialysis-unit. Blood flow is set at 2 ml/min/kg body weight, dialysate flow 1 ml/min. (**B**) 75 fiber dialyzer unit (for details see Table 1) (image produced by B(GH) Janssen).

To establish extracorporeal blood flow, two indwelling catheters were placed; one in the left femoral artery as to enable the blood supply towards the dialyzer unit, while a second indwelling catheter was placed in the left femoral vein to allow the blood to return into the systemic blood circulation (Fig. 1A).

Once these catheters were securely placed and connected to the dialyzer unit, the hemodialysis procedure was initiated, using an extra-corporeal peristaltic pump (P720, Instech Lab., Plymouth Meeting, PA, USA), with a single-lumen tubing (FL-093S-LL, Instech Lab., Plymouth Meeting, PA, USA).

### Surgery-muscle microvasculature

Microvascular observations were performed on EDL muscle as previously described^33^. Subsequently, the tissue is left for an acclimatization period of 30 min before the baseline intravital microscopic observations are made. Observations were made using an inverted microscope (Nikon Eclipse-Ti, Nikon Instruments, Melville, New York, USA), using an adapted microscope stage. The EDL tissue was trans-illuminated (100 W xenon light-source, PTI LPS 220, Horiba Scientific, Piscataway NJ, USA), combined with an optical light guide (Thorlabs, Newton, NJ, USA) and 400–550 nm bandpass filter to prevent any tissue damage related to both the UV and IR light emanating from the xenon light source. An additional filter, (450 nm/20 nm band-pass filter; 450BP20; Omega Optical, Brattleboro, VT, USA), was placed directly in front of the camera to enhance the visualization of red blood cells (RBC).

Intravital microscopic images (1200 × 1920 pixels; 16 bit) of the EDL microcirculation were acquired using a multispectral multi-camera imaging system (MSMC-23-1-A, Spectral Devices Inc., London, Canada), and subsequently stored on a hard drive for later off-line analysis.

### Micro-dialyzer unit

The microdialyzers used in the experiments were assembled using conventional polysulphone fibers collected from existing dialyzers (FX 600 Helixone, Fresenius, Canada) and placed in a polycarbonate tubular housing (Fig. 1B and Table 1). For each experiment a new micro-dialyzer was produced according to these specifications.

**Table 1:** Technical specifications of the micro-dialyzers used in the experiments.

Material outer housing	PMMA
Length	150 mm
Diameter (internal)	6.4 mm
Number of microfibers	75
Material	Polysulphone
Fiber internal diameter	210 µm
Internal volume dialyzer fibers	260 µl
Total internal volume dialyzer	590 µl
Active length^a^	100 mm
Total effective membrane exchange area	49.5 cm^2^
Dialysate flow	approx. 1 ml/min
Dialyzer blood flow	2 ml/min kg
Total extracorporeal priming volume	2.28 ml

All components have been built and designed in-house, polysulphone fibers were harvested from conventional clinical hemodialyzers.

^a^Length of dialyzer fibre section involved in fluid/solute exchange.

### In-vivo hemodialysis combined with intravital microscopy

#### In-vivo dialysis

Prior to the in vivo dialysis procedure, the dialyzer and all connected fluid lines were thoroughly flushed and filled with freshly prepared dialysate fluid. The total volume of the extracorporeal circulation (i.e., dialyzer unit and catheters) was 2.28 ml. Simultaneous dialysate flow (countercurrent to the blood flow in the microfibers), was established using two separate, peristaltic infusion pumps (Sigma Spectrum V8 Infusion System, Baxter, Deerfield, Ill, USA). Pump rates in both dialysis fluid pumps was set at approximately 1 ml/min. Dialysate pump rates were adjusted based on the baseline systemic hematocrit, to ensure an euvolemic HD procedure.

Since approximately 7% of the bodyweight in rats can be attributed to blood volume^34^, we estimate that the systemic blood volume of the animals in our study varies between 17.5 and 21 ml. Consequently, during the hemodialysis procedure, 11–14% of the rat’s systemic blood will be redistributed over the total extracorporeal volume of the mini-dialyzer (2.28 ml).

Although rats appear to be relatively resilient to changes in systemic blood volume either during hemorraghic shock^35,36^, or cardiopulmonary bypass and extracorporeal membrane oxygenation^37–39^, involving significantly larger extracorporeal volume than used in our investigations. However, it cannot be excluded that in our experiments the additional volume of the fully primed extracorporeal circuit can exert a cardiovascular effect during the HD procedure. As such, we included a SHAM procedure, in which a bypass with the same internal volume as the mini-dialyzer (290 µl) is used alongside the min-dialyzer for 1 h, after which blood flow is switched to be pumped through the dialyzer. This allows the animal to become physiologically adjusted to a additional extracorporeal fluid volume, as well as and minimizes the effect of a volume change during HD, since this would only involve the internal volume of the mini-dialyzer.

During the HD procedure, blood is pumped through the dialyzer fibers for 2 h (pump rate: 2 ml/kg/h).

Subsequently blood samples (250 µl) were taken at baseline, after 1 h sham and at 1 and 2 h during HD. Baseline and sham blood samples were taken directly from the dialyzer inflow, while later samples were taken from both the inflow as well as the outflow of the dialyzer unit.

Samples were analyzed using a VetStat point of care blood gas analyzer (VetScan i-STAT-1 Handheld Analyzer, Abaxis, Union City, CA USA) in combination with an iStat Chem8+ cartridge (Abbott, Princeton, NJ USA) respectively, which allow to measure electrolytes (Na^+^, K^+^, Cl^−^, ionized Ca^2+^), hematocrit and hemoglobin, and Urea (BUN) and Creatinine (Crea) in the collected blood samples. An equivalent volume of sterile dialysate fluid was injected to compensate for the change in blood volume. Baseline blood samples were taken during 13 HD experiments; due to unexpected clotting in the extracorporeal dialyzer circuit 11 blood samples were taken at sham and the 1-h HD timepoint, while 9 samples were taken at the 2-h HD timepoint.

#### Intravital microscopy during hemodialysis.

In this study we describe the ability to perform an HD procedure combined with an IVM procedure. Successful IVVM observations were performed in 10 animals during baseline and sham. Some animals were lost during the HD procedure, therefore, at the 1-h and 2-h HD procedure observations were successfully made in 8 and 7 animals respectively. For this, the muscle was surgically prepared and placed under the microscope. After a 30-min acclimatization period, a baseline observation was made from the microvasculature in the muscle, and a selection is made of several adjacent fields of view (FOV). During the following sham and HD procedure, each pre-selected FOV was repeatedly microscopically investigated (once each hour), which allowed to observe the change in perfusion index (i.e., the number of perfused microvessels) during the experimental procedure.

#### Video analysis

Captured IVVM images were stored on a hard drive for later off-line analysis; all intravital observations consisted of a recorded image sequence of 60 s (framerate: 30 frames per second). All images were processed using the in-house written Matlab based software, (Matlab 2020a, the Matworks Inc, Natrick, MA USA; https://www.mathworks.com).

This approach, partially based on algorithms as described earlier^40^, allows identification of vessels that are actively perfused by flowing RBC; by registering the absolute value of light intensity changes in the associated pixels due to the movement of the RBC. Adding up these values, creates (sum of absolute intensity differences; SAD) images, which visualize actively perfused microvessels. Since the same FOV is observed at subsequent time points, it is possible to identify the change over time in the number of perfused blood vessels. To quantify the number of perfused vessels, the subsequent images were super-imposed, ensuring that for each observed FOV the same tissue area is observed, while final assessment of tissue perfusion using a recently developed two-step machine learning algorithm, capable to identify which vessels segments in the observed microvasculature are actively perfused. In short, this algorithm analyses video data by processing the SAD image in relation to am 10 × 10 grid and assessing whether the intersections between grid and the vascular geometric structure are associated with vascular blood flow. For this, the algorithm analyzes each individual intersection with the associated recorded video to assess if blood flow is present or not. Not only does this significantly expedite the analysis of the large video data sets associated with intravital video microscopy, it also ensures a more consistent analysis of the video data compared to a manual analysis step^41^.

### Statistical analysis

Statistical testing was done with GraphPad Prism (Prism 9 for macOS (Version 9.2.0 (283), GraphPad Software, San Diego, California USA, http://www.graphpad.com). All hemodynamics (mean arterial blood pressure and heart rate) are all analyzed either using the Ordinary one-way Anova (Dunnet correction for multiple comparisons) for unpaired data, or the mixed-effect analysis (Dunnet correction for multiple comparisons) for paired data. The number of grid-points indicating vessel-perfusion, are analyzed using the Ordinary one-way Anova (Dunnet correction for multiple comparisons). Blood chemistry values were analyzed using a one-way Anova (Bonferoni correction for multiple comparisons).

## Results

### Intravital microscopy

**Figure 2:**
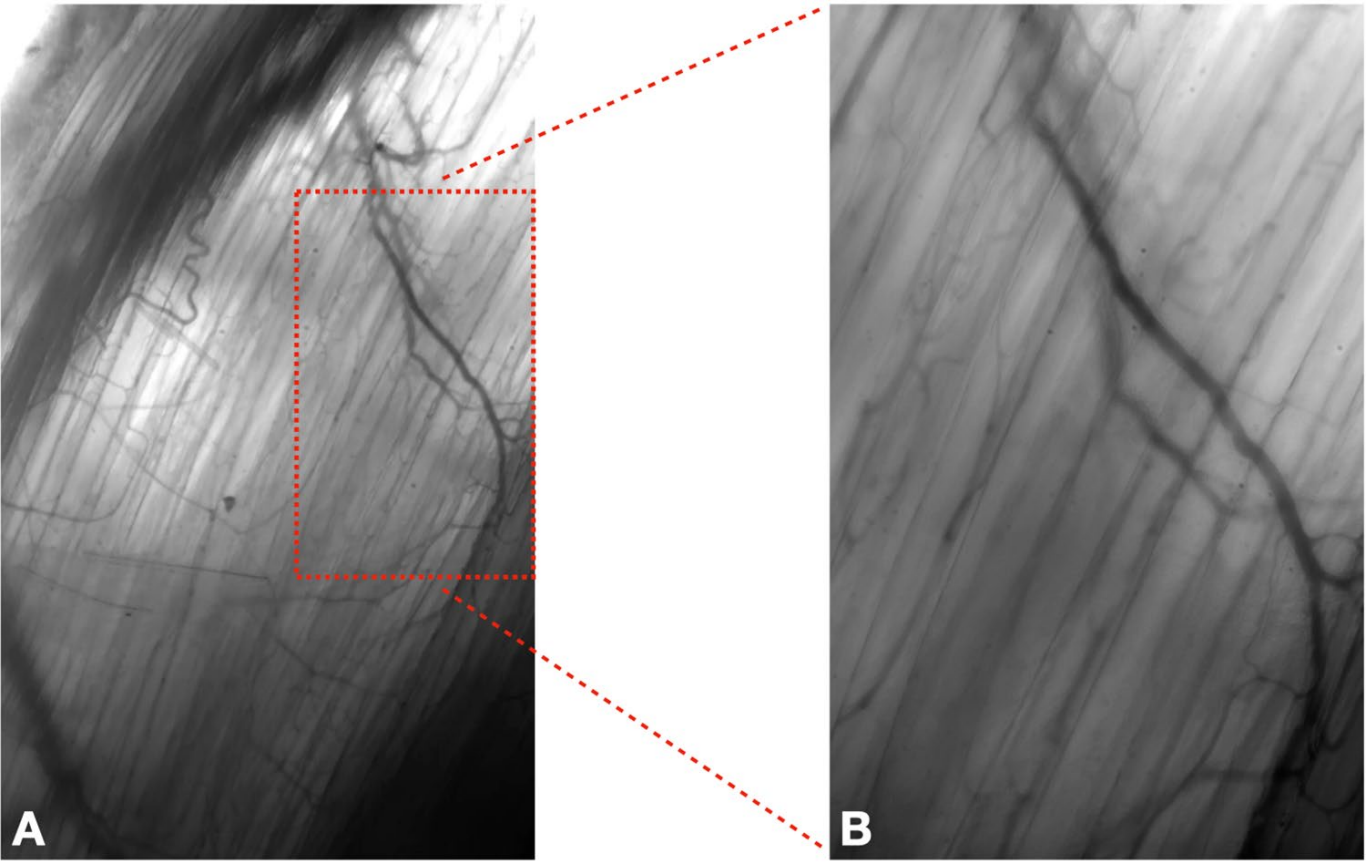
Video still images from IVVM observation of the EDL microvasculature. These images are acquired at baseline, before the start of the HD procedure. The cut-out in (**A**) represents a part of the total field of view (233 µm × 373 µm) shown in (**B**) (93 µm × 149 µm) (see the associated videos in supplemental data: S1-Fig. 2A and S2-Fig. 2B)” (image produced by B(GH) Janssen).

Figure 2 shows still images of a microscopic observation of the EDL muscle microvasculature in the rat during a baseline observation, just prior to the start of the HD procedure (see Supplemental video data). These results show the technical feasibility to observe the microvasculature during a HD procedure, and functionally analyse microcirculatory blood flow.

**Figure 3:**
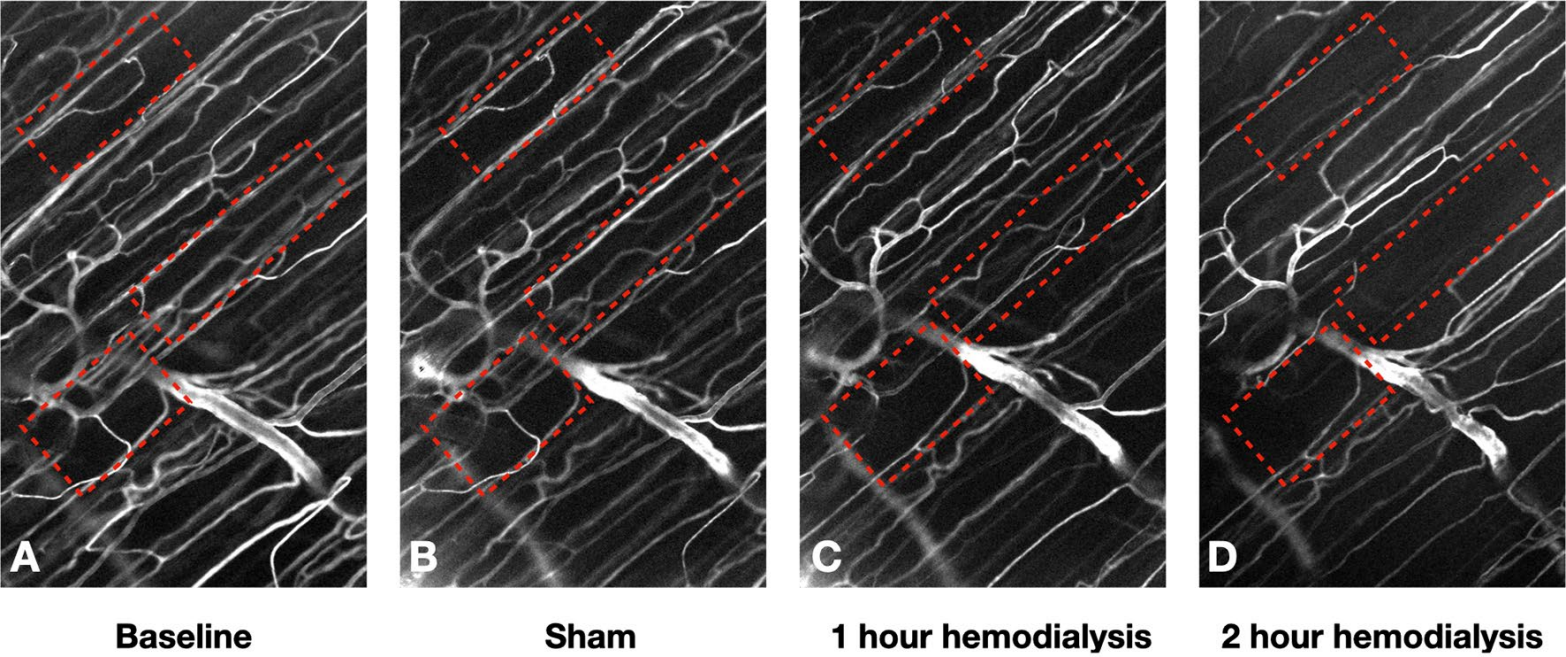
Tissue perfusion during hemodialysis. SAD images from an IVVM observation of the EDL microvasculature at different timepoints during HD (tissue area of 233 µm × 373 µm). (**A**): Baseline SAD image. (**B**): SAD image at 1 h sham procedure. (**C**): SAD image at 1 h hemodialysis procedure. (**D**): SAD image at 2 h hemodialysis (see the associated inverse videos in supplemental data: S3-Fig. 3A, S4-Fig. 3B, S5-Fig. 3C and S6-Fig. 3D) (image produced by B(GH) Janssen using Matlab 2020a, the Matworks Inc, Natrick, MA USA; https://www.mathworks.com).

Figure 3 depicts SAD images showing how tissue perfusion changes during a HD procedure. By observing the same tissue area at different time points during a HD procedure, and process the captured video images to visualize the geometry of the perfused vessel structure, it is possible to assess the change in tissue perfusion during HD. While during baseline observations the tissue is well perfused (see Supplemental video data), at later time points during the HD procedure, microvascular perfusion in a significant portion of the observed tissue significantly impaired. The red rectangles in the SAD images (Fig. 3A–D), representing the same tissue areas in the EDL muscle, show that during the HD procedure less microvessels are actively perfused and remaining tissue perfusion is more sluggish compared to control observations (see Supplemental video data).

**Figure 4:**
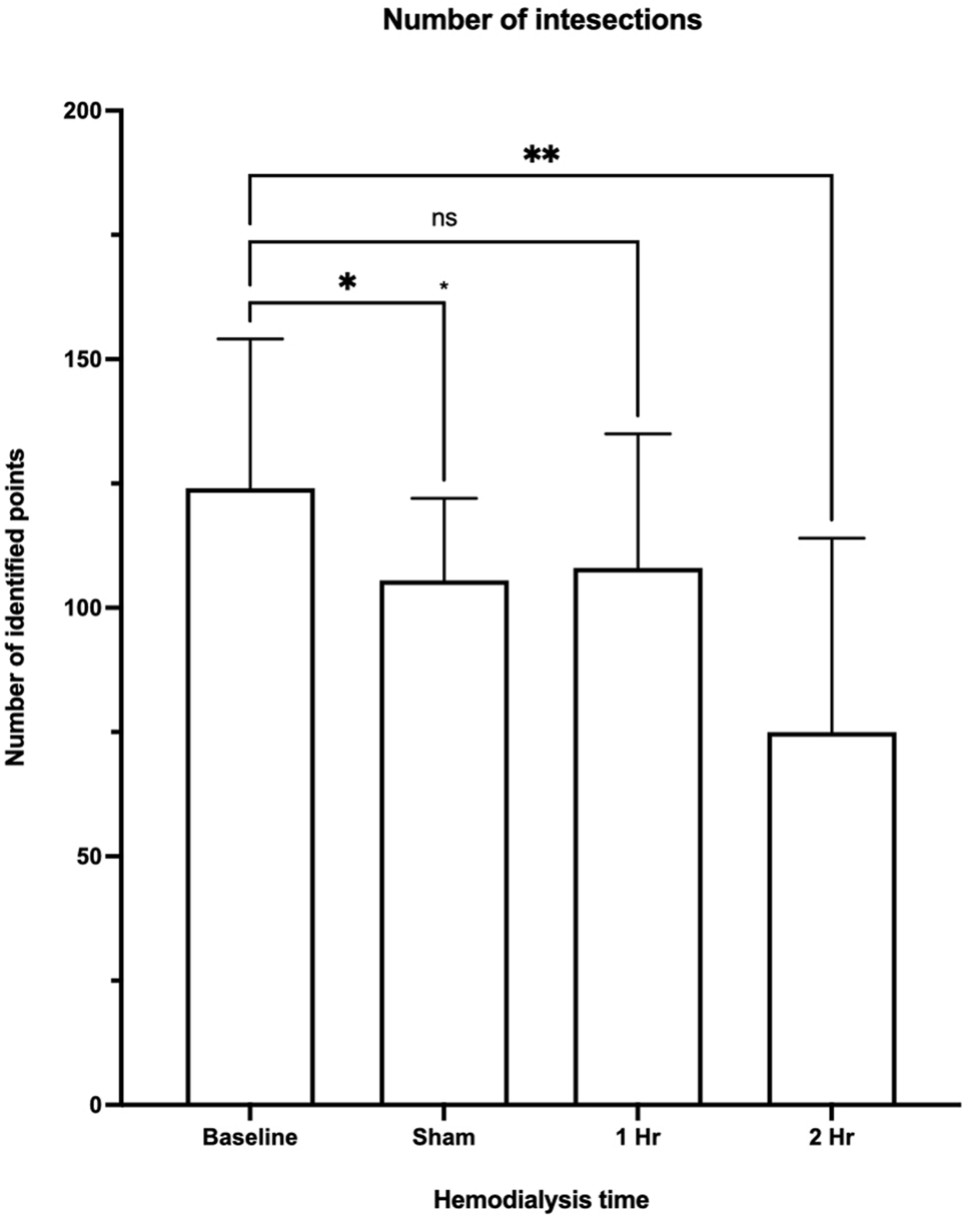
Tissue perfusion of the EDL microvasculature during HD as indicated by the number of grid intersections at different timepoints. Compared to baseline, the number of grid intersections was significantly reduced in the sham group and after a 2-h HD procedure (0.0192 and 0.0035 respectively; Ordinary one-way Anova with Dunnet correction for multiple comparisons) (image produced by B(GH) Janssen using; Prism 9 for macOS (Version 9.2.0 (283), GraphPad Software, San Diego, California USA, http://www.graphpad.com).

Further functional analysis of the intravital microscopic videos using two-step algorithms based on a recently developed machine learning algorithm, capable to identify which vessels segments in the observed microvasculature are actively perfused. Analysis of the video data shows that microvascular perfusion is significantly reduced during the HD procedure. Intravital microscopic observations in 10 animals showed that the number of intersections compared to baseline (n = 47 FOV; mean: 139; range 16–304), indicating the level of tissue perfusion, had significantly reduced during the 1-h sham procedure (n = 46 FOV; mean: 104, range: 16–188), and 2-h hemodialysis procedure (n = 23 FOV; mean: 84; range: 5–229) (respective p-values; 0.0192 and 0.0035; Ordinary one-way Anova with Dunnet correction for multiple comparisons), while compared at 1-h hemodialysis (n = 35 FOV; mean: 119; range: 13–336), no significant difference with baseline was observed (Fig. 4).

### Hemodynamics

**Figure 5:**
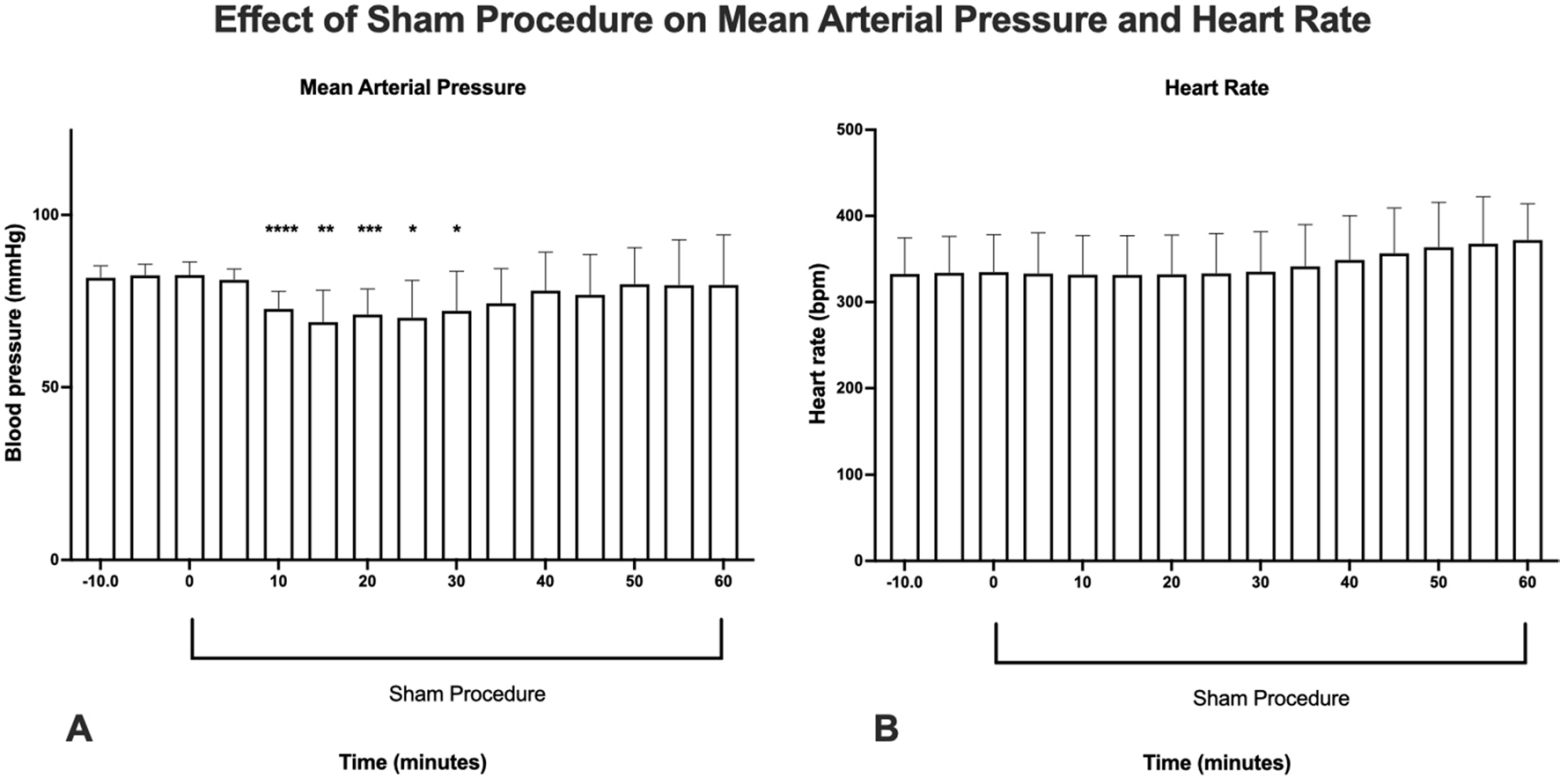
Effect of a 1-h sham procedure on: (**A**) mean arterial pressure and (**B**) heart rate. Sham was started at (timepoint 0 min) and recorded continuously during the experiment (n = 9 animals; mixed-effect analysis with Dunnet correction for multiple comparisons. (*): p < 0.05, (**): p < 0.01), (***): p < 0.001 (****): p < 0.0001 (image produced by B(GH) Janssen using; Prism 9 for macOS (Version 9.2.0 (283), GraphPad Software, San Diego, California USA, http://www.graphpad.com).

Analysis of the hemodynamic data showed that the sham procedure resulted in a moderate and transient reduction in mean arterial blood pressure (Fig. 5A). Compared to baseline t = 0 min, MAP (mean: 82.5 mmHg, range 74–87.3 mmHg) was significantly lowered at 10 min (mean: 72.7 mm Hg; range 64.2–78.5 mmHg), 15 min (mean: 68.9 mm Hg; range 46.9–57.8 mmHg), 20 min (mean: 71.1 mm Hg; range 57.8–80.1 mmHg), 25 min (mean: 70.2 mm Hg; range 46.3–81.5 mmHg) and 30 min(mean: 72.2 mm Hg; range 47.5–83.0 mmHg) into the HD procedure (p-values: < 0.0001, 0.0014, 0.0005, 0.0152, and 0.0465 respectively; mixed-effect analysis with Dunnet correction for multiple comparisons), while MAP recovers back to baseline well before the start of the HD procedure. Heart rate during baseline, ranged from 234 to 381 bpm (mean 335 bpm) and was not significantly affected during the 1-h sham procedure (Fig. 5B).

**Figure 6:**
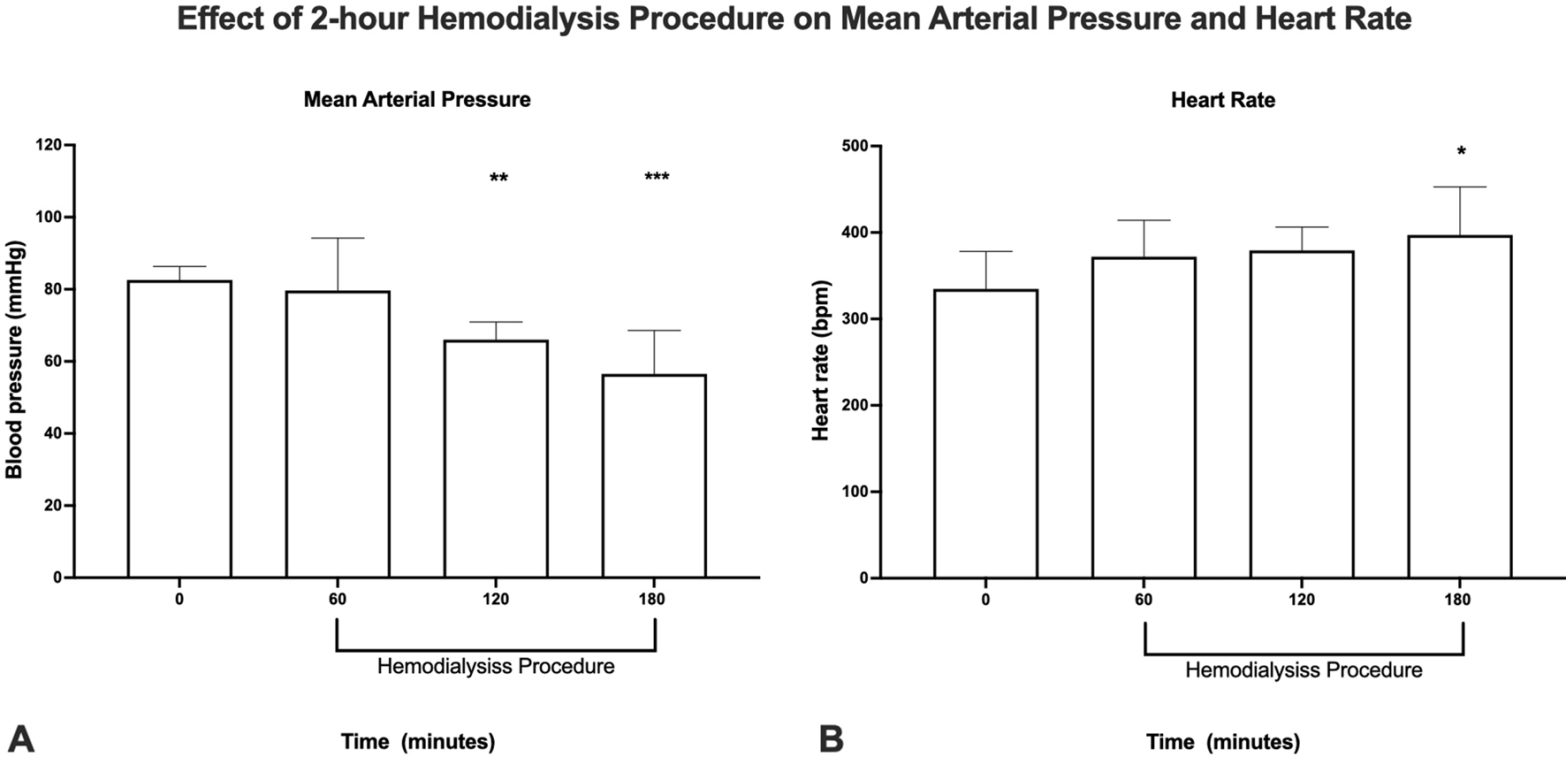
Effect of total experimental hemodialysis procedure on: (**A**) mean arterial pressure and (**B**) heart rate. Hemodialysis started following the sham procedure (timepoint 60 min) and recorded continuously during the 2-h hemodialysis. (n = 9 animals; Ordinary one-way Anova with Dunnet correction for multiple comparisons; (*): p < 0.05, (**): p < 0.01), (***): p < 0.00. (Image produced by B(GH) Janssen using; Prism 9 for macOS (Version 9.2.0 (283), GraphPad Software, San Diego, California USA, http://www.graphpad.com).

In 9 animals we were able to successfully monitor MAP and heart rate during the complete HD procedure. The results show that following the 1-h sham procedure blood pressure (Fig. 6A) dropped significantly from baseline (mean: 82.5 mmHg, range: 74 to 87.3 mmHg) to (mean: 66.0 mmHg, range: 60.8 to 72.6 mmHg) and (mean: 58.5 mmHg, range: 39.7 to 69.4 mmHg) after an HD procedure of respectively 1 and 2 h (p-values; 0.0126 and 0.0001 respectively; One-way Anova with Dunnet correction for multiple comparisons). Heart rate showed a significant increase after a 2-h HD procedure; from 335 bpm (range: 234 to 381 bpm at baseline, to 397 bpm (range: 342 to 499 bpm); p-value: 0.0291 (One-way Anova with Dunnet correction for multiple comparisons).

Heart rate showed a significant increase after a 2-h HD procedure (Fig. 6B); from 335 bpm (range: 234 to 381 bpm at baseline, to 397 bpm (range: 342 to 499 bpm); p-value: 0.0483 (Kruskal–Wallis test with Dunn’s correction for multiple comparisons).

### Blood analysis

Since for this study only healthy animals with intact kidney function were used, initial mean baseline levels of the blood solutes (Table 2) were all within the normal range reported for healthy rats^42^. The dialysate fluid used in our experiments is based on a standard composition used in clinical practice (Table 3). Analysis of the blood samples taken at different timepoints during the HD procedure show that the sham procedure resulted in significantly increased levels of K+ and BUN, while Crea levels were also elevated, yet not statistically significant. However, during the procedure we find that systemic K+, BUN and Crea levels, as represented by pre-dialyzer plasma levels, significantly increased when compared to their respective baseline values, while glucose levels were significantly reduced likely due to the lack of glucose in the dialysate. The mini-dialyzers effectively lowered plasma levels of K+ BUN, Crea and glucose during passage through the mini-dialyzer at 1 h and 2-h HD.

**Table﻿ 2:** Blood analysis (Vetscan Chem8+).

	Baseline (n = 13)	Sham (n = 11)	Dialysis (1 Hr) (n = 11)		Dialysis (2 Hr) (n = 9)	
			Pre-dialyzer	Post-dialyzer	Pre-dialyzer	Post-dialyzer
Ca^2+^ (mmol/L)	1.22 (1.12–1.27)	1.12 (1.14–1.28)	1.17 (1.07–1.29)	1.13 (1.05–1.26)	1.15 (1.06–1.28)	1.12 (1.04–1.24)
Na (mmol/L)	138 (139–140)	137 (135–138)	137 (33–1139)	136 (133–138)	136 (135–137)	135 (133–138)
K (mmol/L)	4.9 (4.5–5.5)	5.5* ^(a)^ (4.7–6.4)	5.5 (3.5–7.6)	3.5** ^(b)^ (3.0–4.1)	6.0** ^(a)^ (5.1–7.2)	3.9*** ^(b)^ (3.5–4.8)
Glu (mg/dL)	298 (137–295)	187 (136–260)	159.0 (84–228)	128.0 ** ^(b)^ (92 -1780)	122.0 * ^(a)^ (56–182)	113.0 (73–155)
TCO2 (mg/dL)	33.1 (31.0–34.0)	31.8 (26.0–34.0)	31.8 (29.0–35.0)	33.3 (28.0–37.0)	30.9 * ^(a)^ (29.0–34.0)	332.2 (27.0–36.0)
HCT (%PCV)	35.7 (31.0–38.0)	34.2 (32.0–40.0)	36.0 (32.0–39.0)	37.8 (28.0–46.0)	34.4 (30.0–39.0)	37.3 (30.0–49.0)
BUN (mg/dL)	22.9 (14.0–29.0)	29.4*** ^(a)^ (23.0–36.0)	31.0* ^(a)^ (9.0–42)	14.2*** ^(b)^ (9.0–20.0)	34.7** ^(a)^ (20.0–42.0)	18.6*** ^(b)^ (11.0–29.0)
Crea (mg/dL)	0.44 (0.20–0.70)	0.59 (0.4–0.90)	0.7 (0.20–1.5)	0.37* ^(b)^ (0.20–0.60)	0.96** ^(a)^ (0.4–1.50)	0.53** ^(b)^ (0.20–1.00)

Mean (range), (n = number of measurements).

One-way Anova (Dunnet correction for multiple comparisons).

(*): p < 0.05, (**): p < 0.01), (***): p < 0.001, (****): p < 0.0001.

(a): compared to baseline sample, (b) compared to pre-dialyzer sample.

**Table 3:** Composition dialysate (freshly made prior to each experiment).

Na^+^	137 mmol/l
K^+^	3.00 mmol/l
Ca^2+^	1.25 mmol/l
Mg^2+^	0.50 mmol/l
Cl^−^	106.5 mmol/l
HCO^3−^	33.0 mmol/l
CH3COO^−^	4.00 mmol/l
Glucose	1.00 g/l
CD	13.745 mS/cm

## Discussion

### General

In this initial study we describe a new small animal model, which allows for an in-vivo investigation of microvascular perfusion during a hemodialysis procedure. Using in-house developed and assembled miniaturized dialyzer units with a very low extracorporeal volume, we can effectively dialyze the small animals and clear in-vivo small molecular solutes directly from the blood.

Our results show that HD is capable to induce a significant disruption in tissue perfusion as observed in the exteriorized EDL tissue in healthy rats during a 2-h hemodialysis procedure, showing that even in the absence of an underlying renal pathology, an euvolemic hemodialysis procedure can directly affect the quality of microvascular perfusion in-vivo.

Our results show that an HD procedure performed with the mini-dialyzers is associated with a significant decrease tissue perfusion when observing the microcirculation in the EDL muscle tissue of healthy animals. The absence of any underlying pathology clearly indicates that a (euvolemic) HD procedure itself directly affects the quality of microvascular perfusion in-vivo.

During an HD procedure, blood is guided from the central blood stream into the dialyzer circuit to mitigate the systemic build-up of excess fluid and metabolic waste materials associated with renal failure. Our studies clearly show that microvascular perfusion is directly affected by a 2-h hemodialysis procedure, showing microvascular perfusion in the selected FOVs, as indicated as the by number of grid intersections representing the number of perfused microvessel segments.

Our results further underline the importance for an in-vivo research model that allows for the direct investigation of the in-vivo correlation between HD and MD. The small size of the animals in this study, facilitates a clear and detailed observation of microvasculature in the EDL muscle, both before, as well as during the HD procedure. So far experimental HD studies have mainly been performed in larger animals like goat^43,44^, pigs^45^, sheep^46^, foals^47^, cats and dogs^48,49^ and have never involved any simultaneous intravital microscopic observation of the microvascular perfusion.

The challenge of producing stable severe chronic kidney failure in larger animals (in contrast to several well-developed methods in rats) combined with the technical complexity of performing a HD in a small animal, has resulted in a paucity in the number of studies which describe the microvascular maldistribution of tissue blood flow during HD in small laboratory animals^50,51^. The technical challenge involves the manufacturing of a mini-hemodialyzer small enough to perform a successful HD in animals under 300 g, as well as the technical skill to simultaneously perform intravital microscopic observations in the tissue.

To our knowledge, this is the first systematic investigation describing the feasibility of a small animal model to study the in-vivo effect of HD on microvascular perfusion. The ability to investigate the development of MD during a HD procedure, allows to study the pathophysiological conditions that cause the HD associated disruption of microvascular blood flow as well as investigate treatments that will mitigate these adverse responses.

### The sham procedure and microvascular perfusion

Our intravital microscopic results show that during the sham procedure, the number of grid intersections representing the level of microvascular perfusion was reduced by approximately 25%, indicating that the additional extracorporeal priming fluid volume reduces tissue perfusion in the observed EDL muscle microvasculature. Although this outcome may directly result directly from a dilution-effect due to the infusion of the priming fluid entering the systemic circulation, however, this notion is not supported by our observation that systemic HCT values, which can be used to assess the fluid balance during HD^52,53^, remain unchanged during the experiment.

The sham procedure was introduced in our model to facilitate the physiological adaptation of the animal to the additional extracorporeal priming volume of the hemodialysis circuit, which comprises approximately 11–14% of the rat's systemic blood volume. The results from our investigations show that this procedure appears to be hemodynamically well tolerated by the animal, inducing only a moderate and transient decrease in mean arterial pressure between 10 and 30 min into the sham procedure. Furthermore, since also heart rate during the sham procedure remains unchanged, we infer that despite changes in the EDL microvascular blood flow, myocardial tissue perfusion is likely not significantly affected by this procedure. Studies have shown that rats appear to be relatively resilient to changes in systemic blood volume either during hemorrhagic shock^35,36^, or cardiopulmonary bypass and extracorporeal membrane oxygenation^37–39^, often involving significantly larger extracorporeal volumes than used in our investigations. Our results are in line with observations from a study describing the effect of a mock model of an extracorporeal membrane oxygenation device in rats, involving an extracorporeal priming volume of 2.5 ml. Similar to our investigations, researchers showed only a limited reduction in mean arterial blood pressure starting shortly after the start of the procedure, while remaining constant during the 8 h observation^38^.

### The hemodialysis procedure and microvascular perfusion

Following the start of HD, we observe a continuing decline of the tissue's microvascular perfusion. Although not statistically significant, after a 1-h HD procedure, microvascular perfusion was found to be below baseline, yet not different compared to the 1-h sham procedure. Continuing the HD procedure shows that between 1 and 2 h, microvascular tissue perfusion continued to deteriorate significantly by approximately 40% compared to baseline value. In addition, we observed a concomitant and consistent decline in MAP and increase in heart rate during the 2-h procedure. Since the HD procedure in our investigations clearly affects the quality of microvascular perfusion of a skeletal muscle, it is likely that perfusion in other microvascular beds e.g., the myocardial microcirculation, would also become affected.

Other investigations have shown that tissue perfusion can be affected by HD, and subclinical perfusion disturbances due to hemodialysis have been reported throughout the entire body^24,54,55^. HD has been shown to induce cardiac stunning in patients due to the maldistribution of myocardial blood flow, which ultimately cause irreversible structural change of the myocardium^25^. Using sidestream-dark-field-imaging, it was recently shown that the sublingual microcirculation was significantly decreased during HD, indicating a direct link between hemodialysis and changes in tissue perfusion resulting in MD. Detailed observations using photon-emission computed tomography revealed that in 70% of patients with no pre-existing coronary pathology, HD would induce myocardial perfusion defects^56^, which due to their subclinical nature, often go undetected^9,57,58^.

It is unclear what causes the intradialytic microvascular defects; although a change in the patient's fluid status can be a contributing factor, it was found that these blood flow disturbances occur early during an HD procedure, and do not coincide with any decrease in blood volume due to ultrafiltration^53^. Research has shown that due to the bio-incomparability of the material of the dialysis membranes and extracorporeal circuit, can result in activation of leukocytes which are subsequently sequestered in the lung microcirculation^59,60^. It has also been shown that formation of microbubbles is a common phenomenon during HD, likely resulting from the fluid dynamic turbulence in the extracorporeal circuit and which can cause damage to tissues by inducing an inflammatory response, complement activation of and coagulation^61^. Since this would directly impede microvascular blood flow in the alveolar microvasculature, it could explain the observed reduction of oxygen uptake^62^ and oxygen saturation levels in hemodialysis patients^63,64^. In most HD patients a decline in blood pressure can found during hemodialysis, which cannot be attributed to the removal of fluid from the central circulation^65^. Studies show that intradialytic reduced oxygen levels are closely associated with intradialytic hypotension^64,66,67^ as well as patient survival^68^. Both interdialytic MD as well as a reduction in oxygen saturation, would affect myocardial function and consequently blood pressure, further exacerbating any developing perfusion disturbances in other tissues throughout the body. It was shown that in patients without any pre-existing coronary pathology, the HD procedure itself diminishes the myocardium's ability to accommodate the required workload by diminishing the coronary flow-reserve^69^. In fact, it was found that severe intradialytic hypotension occurs in 20–30% of patients undergoing HD^70–72^, likely resulting from a fluid imbalance due to ultrafiltration, leading to a hypovolemic state and a reduction of cardiac output and overall tissue perfusion^65^. Studies indicate that dialysate cooling effectively reduces the incidence of the intradialytic hypotensive episodes and improves the hemodynamic tolerability of the HD procedure. Cooled dialysate will lower the patient’s body temperature, resulting in an autoregulatory vasoconstrictive response in the peripheral and cutaneous circulation, and improvement of cardiac output and hemodynamic stability during HD^73^. However, the animal’s body core temperature (as measured continuously using a rectal probe during the procedure) is maintained at 36.5 °C, and although the dialysate is kept at ambient room temperature (24 °C), the animals show no signs of hypothermia. It indicates that in our set-up any transient cooling of blood flowing through the mini-dialyzer, does not affect the core body temperature of the animal. Since in our investigations the fluid status of the animals remains unchanged, the changes in blood pressure cannot be attributed to a hypovolemic state, but more likely point to a bioincompatible interaction between the membrane material and the blood flowing through the dialyzer fibres. Studies in pigs show that a selective extracorporeal cooling down to 20 °C during hemofiltration can be done safely, without affecting blood coagulation or leukocyte activation^74^, in fact some investigations suggest that specific cooling of the hemodialysis filter may improve the membrane’s biocompatibility^75,76^.

The repetitive nature of the HD procedure, ensures that these blood flow disruptions may result in a recurring accumulative ischemic injury in the tissue and ultimately leading to irreversible changes in the affected organs. Whereas MD is a systemic condition it can also affect other microvascular beds throughout the body, and it is therefore reasonable to assume that changes observed in the cardiac microvasculature, may also occur in other anatomical locations. Since the microvascular blood flow is critical for oxygen delivery to the tissue, any disruption would directly affect tissue metabolism and function.

Such a maldistribution of blood flow could explain why in our experiments renal filtration appears to become less efficient during HD. While in the heart this would result into cardiac and hemodynamic failure and arrhythmia, in the brain and liver ischemic injury would lead to cognitive impairment^77^ and a reduced toxin clearance^78^, respectively. Furthermore, it was found that a hemodialysis procedure affects the renal functionality, ultimately resulting in a decline in residual renal function^79^.

Although both Crea and BUN are effectively cleared from the blood when passing the mini dialyzer, during the in-vivo HD procedure both metabolic waste products significantly increase during the procedure, although plasma levels are still within normal range^42^. These results show that even in healthy animals, renal filtration and functionality are significantly affected by the HD procedure. Moreover, since even the HD procedure itself can result in a reduction of renal perfusion and subsequent filtration, it is possible that this would be the reason for the increase of levels of Crea and BUN in our studies. Eventually, if kidney function would become affected and less efficient since the recurrent reduction of renal blood flow could ultimately result in tissue injury and off-set any residual clearing capacity^79^. Interestingly, our results also suggest that some microvascular beds may be more susceptible to HD -associated circulatory disruptions; during the sham procedure plasma levels of BUN and Crea significantly increase, suggestive for a less effective circulatory perfusion and renal clearance, while cardiac function reflected in MAP and heart rate are not affected.

### Limitations of the study

Although this investigation’s primary goal was to corroborate the feasibility of an experimental model that allows to directly investigate microvascular tissue perfusion in tissue during an HD procedure, the results in our study also clearly show how this procedure affects tissue perfusion. Since only healthy animals were used in this investigation, the results can only be interpreted as to the effect of HD in animals with normal kidney function, without any confounding effects of an underlying renal pathology. More studies in animal models with induced kidney failure are needed to further appreciate the impact of disease on HD associated MD.

Although the intravital microscopic observations were made in the EDL, a skeletal muscle in the hind limb of a rat, the results in our study also indicate that other microvascular beds may be affected as well. It is important to note that the machine learning algorithm used to assess the overall maldistribution of blood flow to assesses the tissue’s overall perfusion^41^, and does not analyze local hemodynamic changes in microvascular vessel segments in the tissue. As such, it cannot be excluded that the observed microvascular disruption later in the HD procedure may be preceded by hemodynamic changes in the individual vessel segments.

### Conclusion

In this study, we present an animal model that allows us to directly investigate the perfusion of the microvasculature during a HD procedure. The versatility of this animal model potentially allows for testing of different membrane materials, treatment protocols, and pharmacological interventions in both healthy and diseased animals. As such, it will benefit both a mechanistic understanding of HD-induced ischemic organ injury as well as allow a pre-clinical evaluation of candidate interventions to reduce HD-associated harm to inform the design and delivery of human-based studies.

## Supplementary Information

Supplementary Information 1.

Supplementary Video S1.

Supplementary Video S2.

Supplementary Video S3.

Supplementary Video S4.

Supplementary Video S5.

Supplementary Video S6.

## Abbreviations

BUN: Blood urea nitrogen
Crea: Creatine
CVD: Cardiovascular disease
EDL: Extensor digitorum longus muscle
FOV: Field of view
HD: Hemodialysis
HR: Heart rate
IR: Infra-red
IVM: Intravital microscopy
MAP: Mean arterial pressure
MD: Microvascular dysfunction
RBC: Red blood cells
SAD: Sum of absolute differences
SPF: Specific pathogen free
UV: Ultraviolet

## References

[1] Sarnak MJ, Levey AS (1999). Epidemiology of cardiac disease in dialysis patients. Semin. Dial..

[2] Bleyer AJ, Russell GB, Satko SG (1999). Sudden and cardiac death rates in hemodialysis patients. Kidney Int..

[3] Tonelli M, Karumanchi SA, Thadhani R (2016). Epidemiology and mechanisms of uremia-related cardiovascular disease. Circulation.

[4] Cozzolino M (2018). Cardiovascular disease in dialysis patients. Nephrol. Dial. Transplant..

[5] Tam-Tham H (2020). Association of initiation of dialysis with hospital length of stay and intensity of care in older adults with kidney failure. JAMA Netw. Open.

[6] McIntyre CW (2009). Effects of hemodialysis on cardiac function. Kidney Int..

[7] Georgianos PI, Sarafidis PA, Sinha AD, Agarwal R (2015). Adverse effects of conventional thrice-weekly hemodialysis: Is it time to avoid 3-day interdialytic intervals?. Am. J. Nephrol..

[8] Polinder-Bos HA (2018). Hemodialysis induces an acute decline in cerebral blood flow in elderly patients. JASN.

[9] Buchanan C (2017). Intradialytic cardiac magnetic resonance imaging to assess cardiovascular responses in a short-term trial of hemodiafiltration and hemodialysis. JASN.

[10] Assa S (2018). Effect of isolated ultrafiltration and isovolemic dialysis on myocardial perfusion and left ventricular function assessed with ^13^N-NH _3_ positron emission tomography and echocardiography. Am. J. Physiol.-Renal Physiol..

[11] Hothi DK, Rees L, Marek J, Burton J, McIntyre CW (2009). Pediatric myocardial stunning underscores the cardiac toxicity of conventional hemodialysis treatments. CJASN.

[12] Slessarev M, Salerno F, Ball IM, Mcintyre CW (2019). Continuous renal replacement therapy is associated with acute cardiac stunning in critically ill patients. Hemodial. Int..

[13] Mahmoud H, Forni LG, McIntyre CW, Selby NM (2017). Myocardial stunning occurs during intermittent haemodialysis for acute kidney injury. Intensive Care Med..

[14] Stehouwer CDA (2018). Microvascular dysfunction and hyperglycemia: A vicious cycle with widespread consequences. Diabetes.

[15] Koç AK (2019). Severe OSAS causes systemic microvascular dysfunction. Clinical evaluation of ninety-eight OSAS patients. Clin. Otolaryngol..

[16] Nelson MD, Wei J, Bairey Merz CN (2018). Coronary microvascular dysfunction and heart failure with preserved ejection fraction as female-pattern cardiovascular disease: The chicken or the egg?. Eur. Heart J..

[17] Bordy R (2018). Microvascular endothelial dysfunction in rheumatoid arthritis. Nat. Rev. Rheumatol..

[18] Rost NS (2018). Diffuse microvascular dysfunction and loss of white matter integrity predict poor outcomes in patients with acute ischemic stroke. J. Cereb. Blood Flow Metab..

[19] Jiang H (2018). Altered macular microvasculature in mild cognitive impairment and Alzheimer disease. J. Neuro-ophthalmol..

[20] De Backer D, Orbegozo Cortes D, Donadello K, Vincent J-L (2014). Pathophysiology of microcirculatory dysfunction and the pathogenesis of septic shock. Virulence.

[21] Secor D (2010). Impaired microvascular perfusion in sepsis requires activated coagulation and P-selectin-mediated platelet adhesion in capillaries. Intensive Care Med..

[22] Singer M (2016). The Third International Consensus Definitions for Sepsis and Septic Shock (Sepsis-3). JAMA.

[23] Groeneveld AB (1991). Maldistribution of heterogeneous coronary blood flow during canine endotoxin shock. Cardiovasc. Res..

[24] Bemelmans RHH (2009). Changes in the volume status of haemodialysis patients are reflected in sublingual microvascular perfusion. Nephrol. Dial. Transpl..

[25] Yeh YC (2017). An observational study of microcirculation in dialysis patients and kidney transplant recipients. Eur. J. Clin. Investig..

[26] Penny JD (2018). Percutaneous perfusion monitoring for the detection of hemodialysis induced cardiovascular injury. Hemodial. Int..

[27] De Backer D, Creteur J, Preiser J-C, Dubois M-J, Vincent J-L (2002). Microvascular blood flow is altered in patients with sepsis. Am. J. Respir. Crit. Care Med..

[28] James J, Tanke HJ (2012). Biomedical Light Microscopy.

[29] Bateman RM, Sharpe MD, Ellis CG (2003). Bench-to-bedside review: Microvascular dysfunction in sepsis–hemodynamics, oxygen transport, and nitric oxide. Crit. Care.

[30] Percie du Sert N (2020). The ARRIVE guidelines 2.0: Updated guidelines for reporting animal research. PLoS Biol..

[31] Li C-X, Zhang X (2017). Effects of long-duration administration of 1% isoflurane on resting cerebral blood flow and default mode network in macaque monkeys. Brain Connect..

[32] Szczesny G, Veihelmann A, Massberg S, Nolte D, Messmer K (2004). Long-term anaesthesia using inhalatory isoflurane in different strains of mice-the haemodynamic effects. Lab. Anim..

[33] Tyml K, Budreau CH (1991). A new preparation of rat extensor digitorum longus muscle for intravital investigation of the microcirculation. Int. J. Microcirc. Clin. Exp..

[34] Lee HB, Blaufox MD (1985). Blood volume in the rat. J. Nucl. Med..

[35] Rönn T, Lendemans S, de Groot H, Petrat F (2011). A new model of severe hemorrhagic shock in rats. Comp. Med..

[36] Troy BP, Hopkins DA, Keay KA (2014). The hemodynamic response to blood loss in the conscious rat: Contributions of cardiac vagal and cardiac spinal signals. Shock.

[37] You X-M (2005). Rat cardiopulmonary bypass model: Application of a miniature extracorporeal circuit composed of asanguinous prime. J. Extra Corpor. Technol..

[38] Umei, N. *et al. Establishment and Evaluation of a Rat Model of Extracorporeal Membrane Oxygenation (ECMO) Thrombosis Using a 3D-printed Mock Oxygenator*. https://www.researchsquare.com/article/rs-139858/v1 (2021) 10.21203/rs.3.rs-139858/v1. 10.1186/s12967-021-02847-w PMC8081007 33910585

[39] Dong G-H (2005). A rat model of cardiopulmonary bypass with excellent survival. J. Surg. Res..

[40] Japee SA, Ellis CG, Pittman RN (2004). Flow visualization tools for image analysis of capillary networks. Microcirculation.

[41] Mahmoud O, El-Sakka M, Janssen BGH (2021). Two-step machine learning method for the rapid analysis of microvascular flow in intravital video microscopy. Sci. Rep..

[42] Kurtz DM, Travlos GS, Travlos GS (2017). The Clinical Chemistry of Laboratory Animals.

[43] Yamagishi N, Oishi A, Sato J, Sato R, Naito Y (1999). Experimental hypocalcemia induced by hemodialysis in goats. J. Vet. Med. Sci..

[44] Wester M (2018). Removal of urea by electro-oxidation in a miniature dialysis device: A study in awake goats. Am. J. Physiol. Renal Physiol..

[45] Hoareau GL (2018). A novel perfusion system for damage control of hyperkalemia in swine. Shock.

[46] Bujok J (2018). Sheep model of haemodialysis treatment. Lab. Anim..

[47] Vivrette S, Cowgill L, Pascoe J, Suter C, Becker T (1993). Hemodialysis for treatment of oxytetracycline-induced acute renal failure in a neonatal foal. J. Am. Vet. Med. Assoc..

[48] Mashita T (1997). Short-term hemodialysis treatment in dogs and cats with total uretic obstruction. Jpn. J. Vet. Res..

[49] Bloom CA, Labato MA (2011). Intermittent hemodialysis for small animals. Vet. Clin. Small Anim. Pract..

[50] Yorimitsu D (2012). Establishment of a blood purification system for renal failure rats using small-size dialyzer membranes. Ther. Apher. Dial..

[51] Vu LH, Kellum JA, Federspiel WJ, Cove ME (2019). Carbon dioxide removal using low bicarbonate dialysis in rodents. Perfusion.

[52] Pstras L, Waniewski J, Wojcik-Zaluska A, Zaluska W (2020). Relative blood volume changes during haemodialysis estimated from haemoconcentration markers. Sci. Rep..

[53] Dasselaar JJ (2008). Haemodialysis is associated with a pronounced fall in myocardial perfusion. Nephrol. Dial. Transplant..

[54] Benhamou Y (2014). Detection of microcirculatory impairment by transcutaneous oxymetry monitoring during hemodialysis: An observational study. BMC Nephrol..

[55] Pipili C (2015). Changes in skeletal muscle microcirculation after a hemodialysis session correlates with adequacy of dialysis. IJNRD.

[56] Singh N, Langer A, Freeman MR, Goldstein MB (1994). Myocardial alterations during hemodialysis: Insights from new noninvasive technology. AJN.

[57] Mohi-ud-din K, Bali HK, Banerjee S, Sakhuja V, Jha V (2005). Silent myocardial ischemia and high-grade ventricular arrhythmias in patients on maintenance hemodialysis. Ren. Fail..

[58] Selby NM, Lambie SH, Camici PG, Baker CS, McIntyre CW (2006). Occurrence of regional left ventricular dysfunction in patients undergoing standard and biofeedback dialysis. Am. J. Kidney Dis..

[59] van Teijlingen ME (2000). In vivo visualization of hemodialysis-induced alterations in leukocyte–endothelial interactions. Kidney Int..

[60] van Teijlingen ME (2003). Haemodialysis-induced pulmonary granulocyte sequestration in rabbits is organ specific. Nephrol. Dial. Transplant..

[61] Barak M, Nakhoul F, Katz Y (2008). Reviews: Pathophysiology and clinical implications of microbubbles during hemodialysis. Semin. Dial..

[62] McGuire S (2021). Cardiopulmonary and metabolic physiology during hemodialysis and inter/intradialytic exercise. J. Appl. Physiol..

[63] Harrison LEA, Selby NM, McIntyre CW (2014). Central venous oxygen saturation: A potential new marker for circulatory stress in haemodialysis patients?. Nephron Clin. Pract..

[64] Rotondi S (2018). Oxygen extraction ratio (OER) as a measurement of hemodialysis (HD) induced tissue hypoxia: A pilot study. Sci. Rep..

[65] Reeves PB, Mc Causland FR (2018). Mechanisms, clinical implications, and treatment of intradialytic hypotension. CJASN.

[66] Mancini E (2017). Intra-dialytic blood oxygen saturation (SO2): Association with dialysis hypotension (the SOGLIA Study). J. Nephrol..

[67] Cordtz J, Olde B, Solem K, Ladefoged SD (2008). Central venous oxygen saturation and thoracic admittance during dialysis: New approaches to hemodynamic monitoring. Hemodial. Int..

[68] Chan L (2017). Intradialytic central venous oxygen saturation is associated with clinical outcomes in hemodialysis patients. Sci. Rep..

[69] Tok D (2005). Impaired coronary flow reserve in hemodialysis patients: A transthoracic Doppler echocardiographic study. NEC.

[70] Boon D, van Montfrans GA, Koopman MG, Krediet RT, Bos WJW (2004). Blood pressure response to uncomplicated hemodialysis: The importance of changes in stroke volume. Nephron Clin. Pract..

[71] Bos WJW (2000). Cardiac and hemodynamic effects of hemodialysis and ultrafiltration. Am. J. Kidney Dis..

[72] Daugirdas, J. T., Blake, P. G. & Ing, T. S. *Handbook of dialysis*. (Wolters Kluwer Health, 2015).

[73] Tsujimoto Y (2019). Dialysate temperature reduction for intradialytic hypotension for people with chronic kidney disease requiring haemodialysis. Cochrane Database Syst. Rev..

[74] Krouzecky A (2011). The safety and efficacy of a new anticoagulation strategy using selective in-circuit blood cooling during haemofiltration–an experimental study. Nephrol. Dial. Transplant..

[75] Otte KE (1997). Heparin-free hypothermal hemodialysis at 20 °C improves biocompatibility. Blood Purif..

[76] Maggiore Q, Enia G, Catalano C, Misefari V, Mundo A (1987). Effect of blood cooling on cuprophan-induced anaphylatoxin generation. Kidney Int..

[77] Tian X (2019). The comparison of cognitive function and risk of dementia in CKD patients under peritoneal dialysis and hemodialysis: A PRISMA-compliant systematic review and meta-analysis. Medicine.

[78] Imai S (2018). Deterioration of hepatic oxygenation precedes an onset of intradialytic hypotension with little change in blood volume during hemodialysis. Blood Purif..

[79] Marants R, Qirjazi E, Grant CJ, Lee T-Y, McIntyre CW (2019). Renal perfusion during hemodialysis: Intradialytic blood flow decline and effects of dialysate cooling. J. Am. Soc. Nephrol..

