# Author Correction: Intravital microscopic observation of the microvasculature during hemodialysis in healthy rats

**DOI:** 10.1038/s41598-022-15101-0

**Published:** 2022-06-27

**Authors:** B. G. H. Janssen, Y. M. Zhang, I. Kosik, A. Akbari, C. W. McIntyre

**Affiliations:** 1 Department of Medical Biophysics, Western University, London, ON Canada; 2 Kidney Clinical Research Unit, Lawson Health Research Institute, London, ON Canada; 3 Imaging Program, Lawson Health Research Institute, St. Joseph’s Health Care, London, ON Canada; 4 Robarts Research Institute, Western University, London, ON Canada; 5 Trauma Research Centre, Fourth Medical Center of the Chinese PLA General Hospital, Beijing, 100048 People’s Republic of China; 6 Intensive Care Unit, Tianjin Nankai Hospital, Tianjin, 300100 People’s Republic of China; 7 Kidney Clinical Research Unit (KCRU), London Health Sciences Centre, 800 Commissioners Rd. East, London, ON N6C 6B5 Canada

Correction to: *Scientific Reports* 10.1038/s41598-021-03681-2, published online 07 January 2022

The original version of this Article contained errors in Table 1, where the data stated for ‘Fiber internal diameter’, ‘Internal volume dialyzer fibers’, ‘Total internal volume dialyzer’ and ‘Effective membrane exchange area’ was incorrect.

The original Table 1 and accompanying legend appear below.

**Table 1:** Technical specifications of the micro-dialyzers used in the experiments.

Material outer housing	PMMA
Length	150 mm
Diameter (Internal)	6.4 mm
Number of microfibers	75
Material	Polysulphone
Fiber internal diameter	180 µm
Internal volume dialyzer fibers	290 µl
Total internal volume dialyzer	540 µl
Active length^a^	100 mm
Effective membrane exchange area	5.9 mm^2^
Dialysate flow	approx. 1 ml/min
Dialyzer blood flow	2 ml/min kg
Total extracorporeal priming volume	2.28 ml

All components have been built and designed in-house, polysulphone fibers were harvested from conventional clinical hemodialyzers.

^a^Length of dialyzer fibre section involved in fluid/solute exchange.

The original Article has been corrected.

