# Supplementary material for: Intravital microscopic observation of the microvasculature during hemodialysis in healthy rats
Source: Sci Rep. 2022 Jan 7;12:191. doi: 10.1038/s41598-021-03681-2 (PMC8741960; doi:10.1038/s41598-021-03681-2)
Supplement: Supplementary file 1 — Supplementary Information 1. [file 41598_2021_3681_MOESM1_ESM.docx]

**Supplementary information**

**Supplemental data**

All data generated and analyzed during this study are included in this published article.

Supplementary information: associated video files:

S1-Fig 2A:

Video of a FOV (233 μm x 373 μm) showing microvascular flow in the EDL muscle at baseline.  *(*[*https://figshare.com/s/19ef3cd66986a0011645*](https://figshare.com/s/19ef3cd66986a0011645)*)*

S2-Fig 2B:

Video of a FOV (93 μm x 149 μm) showing microvascular flow in the EDL muscle at baseline.

*(*[*https://figshare.com/s/79a43b7f42047732be13*](https://figshare.com/s/79a43b7f42047732be13%20)*)*

S3-Fig 3A:

Inverse video of a FOV (233 μm x 373 μm) showing microvascular flow in the EDL muscle at baseline. To emphasize the magnitude of microvascular flow in the tissue, the videos are presented using the inverse processed images of the captured microscopic images.

*(*[*https://figshare.com/s/66f088a57f6068c39718*](https://figshare.com/s/66f088a57f6068c39718)*)*

S4 Fig 3B:

Inverse video of a FOV (233 μm x 373 μm) showing microvascular flow in the EDL muscle at baseline. To emphasize the magnitude of microvascular flow in the tissue, the videos are presented using the inverse processed images of the captured microscopic images.

*(*[*https://figshare.com/s/8cad9f57199689c5f514*](https://figshare.com/s/8cad9f57199689c5f514)*)*

S5 Fig 3C:

Inverse video of a FOV (233 μm x 373 μm) showing microvascular flow in the EDL muscle at baseline. To emphasize the magnitude of microvascular flow in the tissue, the videos are presented using the inverse processed images of the captured microscopic images. *(*[*https://figshare.com/s/ebee9dba5a4630521c0e*](https://figshare.com/s/ebee9dba5a4630521c0e)*)*

S6-Fig 3D:

Inverse video of a FOV (233 μm x 373 μm) showing microvascular flow in the EDL muscle at baseline. To emphasize the magnitude of microvascular flow in the tissue, the videos are presented using the inverse processed images of the captured microscopic images. *(*[*https://figshare.com/s/247efe6d4373063ccb32*](https://figshare.com/s/247efe6d4373063ccb32)*)*
